# Recruiting participants for ergonomic research using self-reported stature and body mass

**DOI:** 10.3233/WOR-220565

**Published:** 2023-12-15

**Authors:** Halil Kılıç, Gerbera Vledder, Xinhe Yao, Willemijn S. Elkhuizen, Yu Song, Peter Vink

**Affiliations:** a Department of Woodworking Industrial Engineering, Faculty of Technology, Muğla Sıtkı Koçman University, Muğla, Turkey; b Faculty of Industrial Design Engineering, Delft University of Technology, Delft, The Netherlands

**Keywords:** Anthropometrics, body mass index, participatory research

## Abstract

**BACKGROUND:**

A valid distribution of key anthropometric parameters among participants is often a perquisite of ergonomics research.

**OBJECTIVE:**

In this paper, we investigated the accuracy of self-reported stature and body mass of the population in the Netherlands.

**METHODS:**

Data from 4 experiments was synthesized where in each experiment, participants self-reported their stature and body mass prior to being measured, of which they were not notified before.

**RESULTS:**

Statistical analysis of 249 records indicated that on average, participants overreported their stature by 1.31 cm and underreported their mass by 1.45 kg. This is especially true for people with a BMI≥25.

**CONCLUSION:**

Two models were proposed to adjust the self-reported stature and body mass for ergonomic researchers in a survey or recruitment. Limitations in using the models are highlighted as well.

## 1 Introduction

In the context of ergonomics research, the anthropometric measurements of participants are often a prerequisite. Self-reported techniques are often used to collect information on the stature, the body mass, and the calculated body mass index (BMI, in kg/m2) during survey and recruiting subjects. In different (online) surveys, the height and body mass can be important factors in evaluating physical activities, perceptions, etc. [1, 2]. For instance, Liu et al. asked the height and the body mass of 90 participants via questionnaires for studying comfort of seats in a staggered configuration [3]. In an online survey designed by Srinivasan et al. on the topic of college students’ personality under the impact of COVID-19 online classes, self-reported height and body mass of 897 subjects were collected as well [4]. To recruit subjects for an experiment, following the requirements and within the practical constraints, researchers often try to ensure a sparse distribution of key measurements, e.g. the stature, the body mass, among the participants to reduce the specificity of the target group and ensure the quality of the research [5, 6]. For instance, in evaluating comfort of an economy seat, researchers tried to select 97 test subjects out of 125 applicants based on the self-reported stature and body mass [7]. Besides, a better estimation of the actual stature and body mass based on self-reported data might accelerate the process of ergonomics experiments and reduce the cost. Measuring anthropometrics consumes valuable time and manpower. Extensive measurements are seen as “tedious and time consuming” [8]. For improving the accuracy, researchers also need to conduct prior-training sessions and repeatable measurements are often taken [9], which both cost extra time and efforts.

Literature supports the theory that the body mass can be underreported and the stature can be overreported in different contexts [10]. For instance, women under-reported their mass by a mean of 0.91 kg in the Health Initiative Observational Study [11], and men overreported their height by 1.22 cm on average in National Health and Nutrition Examination Survey [12]. However, there are also reports indicating that such self-reported body mass and stature were accurate enough in different studies, e.g. Hodge et al. suggested that BMI computed from self-reported weight and height is a valid measure in men and women across different socio-demographic groups [13]. Kee et al. also reported the high correlation between self-report mass and stature with direct measurements in the adolescents group [14].

The accuracy of the self-reported stature and body mass is influenced by many factors, e.g., the types of survey, the social-demographic contexts, the sex. The existence of self-stigma regarding the body mass [15] could influence the subjective ratings in ergonomic studies as well. In research on comparing three different survey data, Flegal et al. found that self-reported height, weight, BMI, and obesity prevalence were not identical across the National Health and Nutrition Examination Survey, the National Health Interview Survey, and the Behavioral Risk Factor Surveillance System, particularly for women [16]. Gugushvili & Jarosz also found that women living in rural areas were likely to overestimate their height [17]. Maukonen et al. also showed that for overweight and obese participants, the bias was generally higher than those of average weight. Regarding cultural background, the bias was larger in North America, while in Asian studies the bias seemed to be lower [10]. For instance, Xie et al. found that the stature was overestimated at an average of 0.42 cm, but there were no significant differences regarding the body mass and blood pressures between self-reported and measured data in Hong Kong [18]. Meanwhile, the stature and the body mass of a subject are not static during the day. Vuvor and Harrison found that the mean stature variation from 7am to 7pm was about 1.61 cm [19]. The average mass fluctuation of a person in a short term, e.g. a day or several days, can also be 1 to 2 kg [20].

While the accuracy, the reliability and the associated factors are still in debate, the self-reported measurements are the easiest, or sometimes the only way, for acquiring the basic anthropometric parameters in the survey and selecting participants in ergonomics research. In this paper, we aim at building models to estimate the actual stature and body mass based on of the self-reported data of adults recruited in the Netherlands in the context of ergonomic research. The outcomes might help ergonomics researchers in using self-reported stature and body mass for survey and recruitment in ergonomics studies.

## 2 Materials and methods

**Fig. 1:**
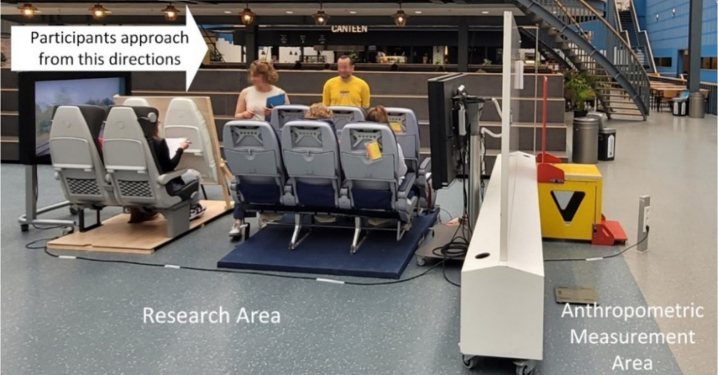
Setup of a typical experiment.

We collected data from 4 ergonomics experiments conducted in the Faculty of Industrial Design Engineering, Delft University of Technology. All experiments focused on evaluating comfort of participants, but in different contexts as: Exp.1) flying with a turboprop aircraft (November, 2021), Exp.2) experiencing jet and turboprops noise (February, 2022), Exp.3) sitting with different postures (March, 2022) and Exp.4) sitting in train and aircraft seats (June, 2022). In those experiments, participants self-reported their stature and body mass prior to being measured, of which they were not informed beforehand. For instance, in Exp.4 an ergonomics research context was created on the topic of evaluating the comfort experience of sitting in train and aircraft seats (see Fig. 1). A large room divider was placed in the middle and anthropometric measurement tools were placed at another side of the room divider. Participants were invited to the experiment in different timespans of a day across 3 weeks. They were instructed to approach from the ergonomics research area where the anthropometric measurement tools were invisible to them. After acquiring the informed consent, participants were asked to fill in a questionnaire about their sex, age, nationality, stature and body mass. Then a researcher led them to another side of the room divider where the basic anthropometric measurements were taken.

All study protocols were approved by the human research ethical committee at Delft University of Technology. Respectively under ID number 1823 (Exp.1), 1953 (Exp.2), 1228 (Exp.3), and 2248 (Exp.4). It is worth mentioning that participants were free to withdraw at any moment of the experiments and his/her data (if any) was destroyed on-site. From the 4 experiments, we collected 76, 15, 28 and 130 valid samples, resulting a total of 249 records. In Exp.1 approximately half of the participants were measured between 08 : 30 and 09 : 30, the other half was measured between 12 : 30 and 13 : 30. During Exp.2 all measurements were made between 13 : 00 and 13 : 30. For Exp.3 measurements were made in the afternoon. Exp.4 started at 09 : 00 and lasted until 18 : 00. G*Power calculation indicated that for identifying small effects (0.3) of paired samples using Wilcoxon signed rank test in a two-tails setup, 154 samples were needed at the power of 0.95. The 249 valid records were analyzed by statistical tools using a self-developed Python program. All participants took off their shoes and jacket and no extra adjustments, e.g. weight of clothing [21], were introduced as interviews of the participants revealed mixed scenarios regarding self-weighting. Based on statistical analysis results, different groups were highlighted.

## 3 Results

Table 1 presents the descriptive statistics of each experiment result as well as a whole. All participants were adults and their mean age is about 28 years. There are high correlations between the self-reported and the measured stature (0.981), and between the self-reported and the measured body mass (0.967). However, people overreported their stature by 1.31 cm on average, and underreported their body mass by a mean of 1.45 kg. Subsequently, the difference of BMIs based on self-reported data and the measured data is about 0.82 kg/m^2^. Regarding individual experiments, Exp.2 and 3 were targeting at the international population and in Exp.1 and 4, the Dutch population was the majority. Though populations differ, Mann-Whitney U test indicated that there was no statistically significant difference (*p* > 0.01) regarding BMIs calculated based on the self-reported data and the measured data between Dutch and international populations. Therefore, all experiment data were combined together in the following analysis.

**Table 1:** Descriptive statistics of the collected data

	Exp. 1	Exp. 2	Exp. 3	Exp. 4	All experiments
Total number	76	15	28	130	249
Gender (female/male)	49/27	9/6	13/15	66/64	137/112
Age	33.42±14.02	26.33±3.66	27.82±2.89	25.15±9.15	28.04±10.87
Nationality (NL/INT)	32/44	4/11	1/27	93/37	130/119
Mass (kg, self-reported)	71.40±12.40	69.87±16.07	62.29±9.98	71.54±12.57	70.36±12.75
Mass (kg, measured)	73.11±13.33	71.73±17.05	62.14±10.66	73.13±12.97	71.81±13.49
Mass difference (kg)	1.71±3.19	1.86±4.45	–0.14±3.33	1.59±3.40	1.45±3.43
Stature (cm, self-reported)	177.31±10.63	171.00±10.45	168.02±6.60	177.19±10.30	175.82±10.50
Stature (cm, measured)	176.24±10.49	169.76±9.28	166.03±6.94	175.87±9.90	174.51±10.28
Stature difference (cm)	–1.06±2.29	–1.24±3.40	–1.99±1.53	–1.31±1.78	–1.31±2.05
BMI (kg/m^2^, based on self-reported data)	22.61±2.74	23.70±3.95	21.99±2.78	22.66±2.54	22.63±2.73
BMI (kg/m^2^, based on measured data)	23.43±3.11	24.64±4.10	22.49±3.24	23.53±2.92	23.45±3.10
BMI difference (kg/m^2^)	0.83±0.95	0.94±1.66	0.50±1.23	0.87±1.17	–0.82±1.15

**Fig. 2:**
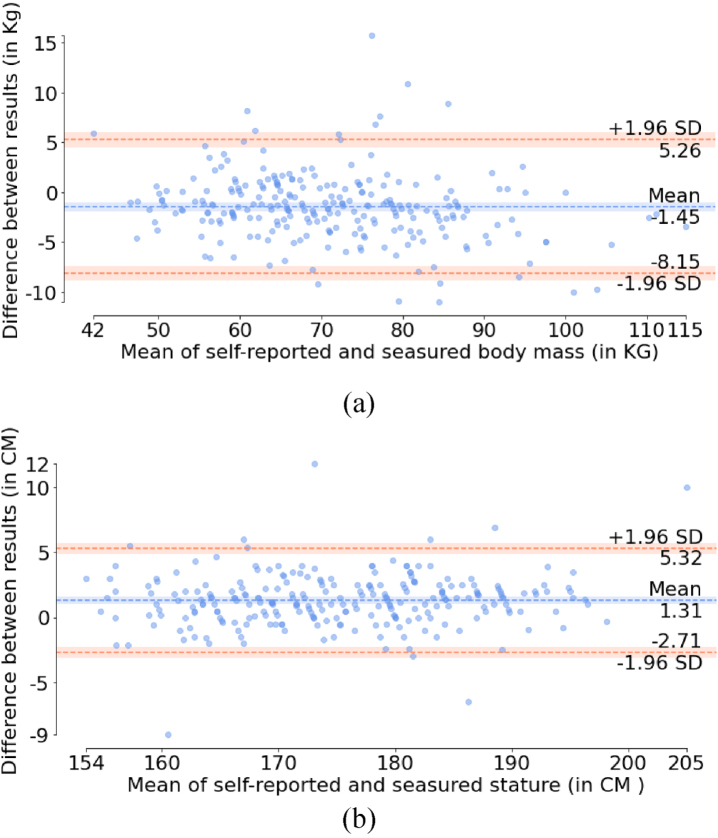
Bland-Altman plots of the self-reported stature (a) and body mass (b) and regarding the means of using the two methods.

Bland-Altman plots (Fig. 2) on the stature and the body mass affirm the differences. The stature difference is 0.74% higher than the mean value while the body mass difference is about 1.93% lower than the mean. Normality test results indicated that the distribution of the differences does not follow the normal distribution. Wilcoxon signed rank test results showed that the differences of the self-reported and the measured stature (*p* < 0.01), body mass (*p* < 0.01) and BMI (*p* < 0.01) are statistically significant.

**Fig. 3:**
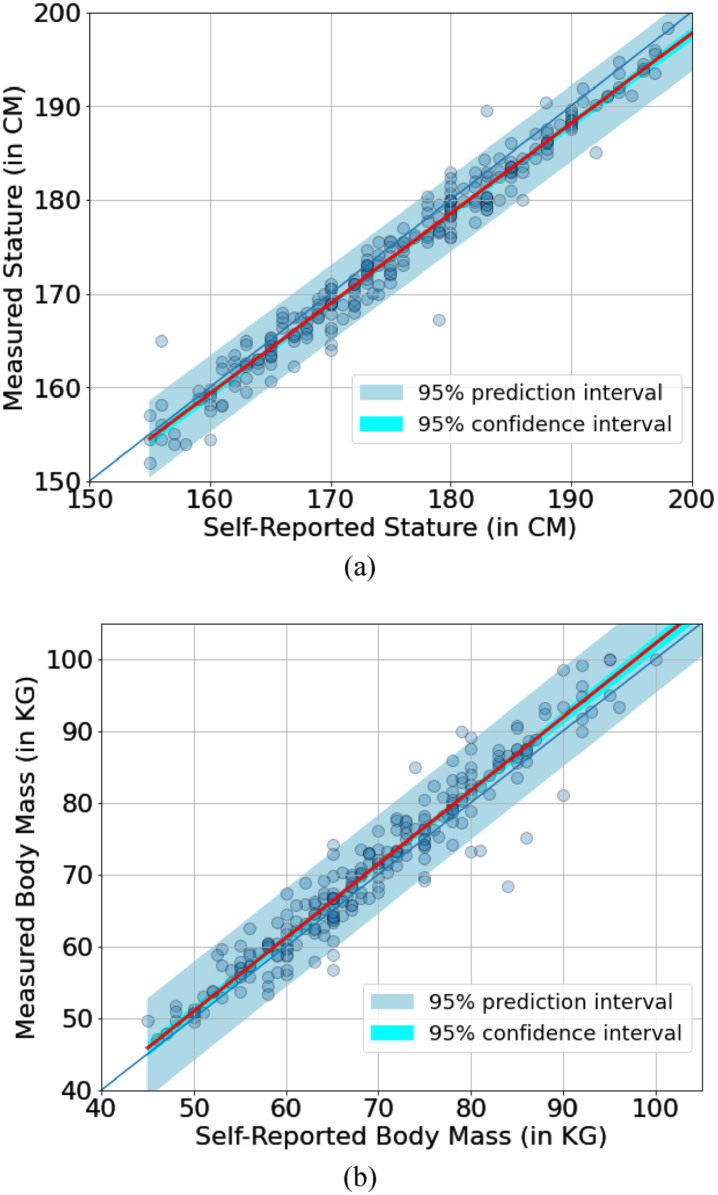
The regression model (red lines) regarding the data.

Figure 3 presents two regression models where the 95% prediction and 95% confidence intervals are highlighted as well. In the model, the measured stature can be calculated from the self-reported stature as:

(1) $$\begin{array}{cc} & \mathrm{Measured}\;\mathrm{Stature}=0.96375*\\ & \;\;\mathrm{Self}\;\mathrm{Reported}\;\mathrm{Stature}+5.36 \end{array}$$

with a root mean square error (RMSE) of 2.24 CM. For the body mass,

(2) $$\begin{array}{cc} & \mathrm{Measured}\;\mathrm{Mass}=1.04469*\\ & \;\;\mathrm{Self}\;\mathrm{Reported}\;\mathrm{Mass}-1.48 \end{array}$$

and the RMSE is 3.12 Kg.

**Fig. 4:**
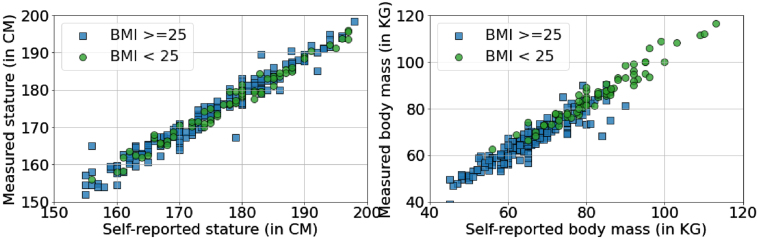
Measured stature and body mass vs the self-reported body mass and stature regarding different BMI groups.

Though all experiments were conducted in the context of ergonomic research, different factors, e.g. sex, nationality and BMI, might have influenced the accuracy of the self-reported stature and the body mass. We categorized the data by sex, nationalities and BMI where for BMI, the Centers for Disease Control and Prevention (CDC) recommendation (BMI < 18.5 is underweight, between 18.5 and 24.9 is healthy, between 25.0 and 29.9 is overweight and larger than 30.0 is obesity) [22] was adopted. Mann-Whitney U test results indicated that there was not statistically significant difference in those groups except the mass difference of the BMI < 25 group (180 subjects, mean=–0.76 kg) was significantly smaller (*p* < 0.01) than the BMI≥25 group (69 subjects, mean=–3.24 kg) as Fig. 4.

**Fig. 5:**
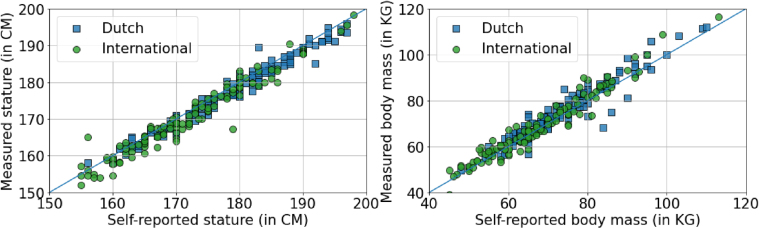
Measured stature and body mass vs the self-reported body mass and stature regarding Dutch & International groups.

There are statistically significant differences of the measured stature and the body mass between the Dutch and the international groups. The mean statue of the Dutch group is 177.79±9.17 cm versus 170.93±10.26 cm of the international group. Regarding the body mass, the values are 75.23±13.31 kg and 68.03±13.75 kg for Dutch and international groups, respectively. However, we did not find statistically significant differences between the self-reported data and the measured data for both groups as shown in Fig. 5.

Literature indicated that sex and BMI are two important factors of the accuracy of the self-reported stature and body mass, we categorized all data using these two criteria as Table 2. It can be found that for the self-reported stature, all groups are within the accuracy of 2 cm where women are slightly better than men (1.02 cm vs 1.55 cm). For the body mass, in general men are slightly more accurate than women (–1.24 vs –1.70 kg), but for the BMI 18.5–24.9 groups, both men and women are quite accurate. The largest difference was observed for men and women with BMI≥25 groups, where the mean body mass differences were found as –2.95 kg and –3.5 kg, respectively. Though the mean difference of women is higher than that of the men, it is not statistically significant (*p* > 0.01). Regarding nationality, Dutch men and women BMI≥25 groups underreported the mass about 2.70 and 3.66 kg, and for the International BMI≥25 groups, the values are 3.50 and 3.89 kg, respectively.

**Table 2:** Differences of self-reported mass and height regarding measurement for different groups

BMI	All groups	<18.5	18.5–24.9	25–29.9	>30
Men				
No.	137	1	92	37	7
Mean Stature	180.37±8.50	n/a	179.81±8.71	182.51±7.52	178.57±8.75
Mean Mass	77.49±13.26	n/a	71.43±8.71	89.13±8.32	101.24±10.80
Mean BMI	23.74±3.26	n/a	22.03±1.61	26.73±1.35	31.68±1.36
Stature Diff.	1.55±2.16	n/a	1.38±2.24	1.92±2.03	1.71±1.90
Mass Diff.	–1.24±3.85	n/a	–0.50±3.94	–3.03±2.79	–2.53±4.03
BMI Diff.	–0.77±1.19	n/a	–0.48±1.07	–1.45±1.05	–1.27±1.71
Stature Diff. in %	0.87% ±1.21%	n/a	0.78% ±1.28%	1.05% ±1.08%	0.93% ±1.05%
Mass Diff. in %	–1.28% ±5.11%	n/a	–0.56% ±5.40%	–3.34% ±2.98%	–2.28% ±3.86%
BMI Diff. in %	–0.90% ±1.56%	n/a	–0.66% ±1.54%	–1.60% ±1.13%	–1.15% ±1.66%
Women				
No.	112	2	85	22	3
Mean Stature	167.34±7.30	173.40±9.90	166.93±7.36	167.79±6.37	171.60±11.75
Mean Mass	64.85±10.11	55.25±6.43	61.32±6.95	75.64±6.58	92.20±12.23
Mean BMI	23.11±2.89	18.34±0.05	21.97±1.66	26.83±1.25	31.22±0.64
Stature Diff.	1.02±1.88	–1.40±1.41	1.02±1.98	1.03±1.47	2.40±0.95
Mass Diff.	–1.70±2.82	–0.25±0.64	–1.13±2.67	–3.50±2.42	–5.53±2.74
BMI Diff.	–0.89±1.10	0.20±0.51	–0.68±1.00	–1.56±1.01	–2.61±0.95
Stature Diff. in %	0.61% ±1.14%	–0.79% ±0.77%	0.62% ±1.21%	0.61% ±0.88%	1.39% ±0.49%
Mass Diff. in %	–2.43% ±4.30%	–0.52% ±1.21%	–1.77% ±4.38%	–4.67% ±3.36%	–5.87% ±2.45%
BMI Diff. in %	–1.31% ±1.67%	0.31% ±0.90%	–1.09% ±1.67%	–2.09% ±1.42%	–2.82% ±0.83%

## 4 Discussion

In this paper, we investigated the accuracy of self-reported stature and body mass regarding the anthropometric measurements within the ergonomics research context in the Netherlands. Experiment results support the declaration that people slightly overreport their stature and underreport their body mass, which is in accordance with some studies in Table 3. This is especially true for people with BMI≥25, where statistically significant difference regarding the self-reported and the measured body mass was identified with a mean difference of –3.24 kg. Compared to a previous study on the Dutch population [21], the difference of the stature and the body mass are slightly larger, which might be that we did not introduce extra adjustments. However, it is in accordance with the review conducted by Maukonen et al. [10], where large BMI population tends to report less on their body mass.

**Table 3:** Comparison of this study and some previous studies

Reference	Stature difference (in cm/%)		Mass difference (in kg/%)		Context	Country
	Men	Women	Men	Women	
[13]	2.08	1.07	–0.726	–1.406	Health Insurance Study	USA
[4]	1.23	0.60	–1.85	–1.40	European Prospective Investigation into Cancer and Nutrition	UK
[14]	1.7	1.1	–0.4	–1.1	The CAESAR Project 1999–2000	Italy, NL and North America
[11]	0.8	0.8	–0.9	–0.8	Amsterdam Health Monitor 2004 Survey	NL
[15]	0.3	2.3	–1.00	–2.8	Survey in a music festival	Australia
[16]	0.7	–0.6	–1.3	–1.4	European Health Examination Survey	12 EU countries
[5]	0.48	0.16	–1.55	–0.88	Cancer Prevention Study	USA
This study	1.55/0.87%	1.02/0.61%	–1.24/–1.28%	–1.70/–2.43%	Ergonomics studies	NL

Although the time of measurements for all experiment is known, the time and date of self-reported measurements are unknown. Vuvor & Harrison found that the daily stature variation of adults aged≥30 years is as high as 1.61 cm [19]. Reilly et al. indicated that the peak variation of the stature can be 1.93 cm or 1.1% of the overall stature [27]. 71% of the height gained during the night was achieved in the first half of the night’s sleep, and over 50% of the height loss in a day happened in the first hour of rising, and 80% was lost within 3 hours of arising. Considering the time of the experiment, e.g. Exp.2 and 3 took place after 3 hours of arising, the main decrease in stature changes already happened. The identified mean difference of the stature is 1.31 cm, which is slightly larger than 50% of the peak variation.

Regarding the body mass, Turicchi et al. found that there are weekly, seasonal and Christmas patterns where within a week, the mean body mass fluctuations are 0.35% [28]. Whigham et al. indicated that the average clothing weight throughout the year was significantly greater in men (1.2±0.3 kg) than in women (0.8±0.3 kg) [29]. Besides, the timing for defecation (median 0.128 kg/cap/day) and urination (median 1.42 L/cap/day) might also influence of the measured body mass [30]. Considering the uncertainty in the self-measurement time and conditions, it can be assumed that for groups with BMI < 18.5, the self-reported body mass is within the fluctuation ranges. However, for BMI≥25 groups, the difference between the self-report body mass and the measurement is larger.

In the measurement results, the mean heights are 180.37 cm and 167.34 cm for men and women, respectively. These are close to the P50 data in the Dutch anthropometric database [31]. The smaller standard deviations of women on the self-reported body mass indicated that women might know their actual body mass better than men, as the self-weight frequency of women is higher than that of men [32, 33].

With the underreported mass, 35% (24 of 69) of the participants in the BMI≥25 group (mean BMI = 25.9) lowered their reported BMI to the range of BMI < 25 (mean = 24.11). The reasons that the self-reported body mass of participants in this group is lower than the measurement might be complicated. Besides sex, nationality, Althubaiti summarized that self-reported bias can also arise from social desirability, recall period, sampling approach, or selective recall these aspects [34]. The accordance with literature indicates that the ergonomics research context has little influence on the bias. For recall period, sampling approach, or selective recall, previous research indicated that the self-weighing frequency of BMI≥85th percentile is even higher than that of the 15th≤BMI<85th percentile group [33]. Social desirability might be the most influential factor on the bias. The body shape is associated with the self-image of a person, and a positive self-image is an important aspect for people to recognize the assets, the limitations and the potentials, and keep positive motivation on tasks and liabilities. For this, people tend to think towards the positive side on their self-image rather than generate it based on facts, e.g. people are more accurate while their focus is on the external observers [35]. This might be more obvious for women, as they are more critical of their body shape [36]. Therefore, participants with larger BMI might want to report less of their body mass to maintain a positive self-image on the body shape, as in most cases the stature is more a nature and more visible than the body mass.

In summary, participants slightly overreported their height and underreported their mass. The stature differences between the self-reported and measured data slightly exceed the daily fluctuation range of a person. The differences of the self-reported body mass and measured results of the BMI < 24.9 group is within the daily fluctuation range. But for the BMI≥25 group, the difference is larger. For ergonomics studies where the anthropometric measurements were not possible, e.g. in survey, in recruitment, it is suggested that the self-reported stature and the body mass can be adjusted using the linear regression models (Eq.1 and 2). In this case, the RMSEs of the differences in the stature and the mass can be reduced from 2.43 cm to 2.02 cm and from 3.71 kg to 3.42 kg, respectively. This is especially useful for the body mass of the BMI≥25 group where the RMSE is reduced from 4.37 to 3.06 kg. In the experiment, when there are constraints regarding both/either manpower and/or time, the proposed model can be used as a backup tool. However, ergonomics researchers shall be aware of the large RMSEs and risks of peak variations in using this technique, as the peak variations of the self-reported statures are –5.5% and 7% and for the self-report body mass, they are –12.8% and 22.9%.

This study has several limitations. Most of the International group participants were studying/working in the Netherlands. We did not find significant differences of self-reported data between Dutch and International groups. Maukonen et al. reported a difference between Asian and American studies, which means a comparison study in other countries might find more social-cultural factors that influence the accuracy of self-reported stature and body mass [10]. Though researchers paid extensive efforts in recruiting. the mean age (28.04 years) of participants was young and the number of participants, especially in the BMI < 18.5 and BMI > 30 groups, was low compared to other studies that were mainly based on national surveys. This prevented the use of advanced modeling tools to make a more accurate estimation based on more parameters, e.g. gender, measurement time in a day, time duration from the last self-measurement. However, the context of the study is ergonomics research, which casts a new view toward the bias of participants in self-reporting their stature and body mass.

## 5 Conclusion

In this paper, we compared the self-reported stature and body mass regarding the measurements in the context of ergonomics research, and try to provide a tool for ergonomic researchers and practitioners for estimating the actual stature and body mass based on self-reported data. Experiment results supported the findings reported in the literature that people slightly overreport their stature and underreport their body mass, especially in the BMI≥25 group. It is suggested that in ergonomic research especially in the survey and recruitment where conducting anthropometric measurement is not possible, or too expensive regarding both manpower and time, researchers can use the proposed models, with the understanding of the limitations, to estimate the actual stature and body mass based on self-reported data, especially for the BMI≥25 group.

## Ethical considerations

All study protocols were approved by the human research ethical committee at Delft University of Technology (ID numbers 1823 (Exp.1), 1953 (Exp.2), 1228 (Exp.3), and 2248 (Exp.4).

## Informed consent

All participants involved in this study signed an informed consent form before participating in this study. The informed consent followed the guidelines of the human research ethical committee at Delft University of Technology and was approved by this same committee.
